# Supplementary material for: Macaque-human differences in SARS-CoV-2 Spike antibody response elicited by vaccination or infection
Source: bioRxiv. 2021 Dec 3:2021.12.01.470697. Preprint. [Version 1] doi: 10.1101/2021.12.01.470697 (PMC8669841; doi:10.1101/2021.12.01.470697)
Supplement: 1 [file NIHPP2021.12.01.470697V1-supplement-1.pdf]

Supporting information

S1 Fig: Enrichment of wildtype peptides varies by group in newly defined epitope regions. The locus numbers are shown on the x axis, and each individual is represented in a different color. (A) Wildtype enrichment by group from AA 526-685, spanning the CTD-N’ and CTD-C’ epitopes. (B) Wildtype enrichment by group from AA 777-855, spanning the pre-FP, FP, and post-FP epitopes. (C) Wildtype enrichment by group from AA 1135-1204, spanning the SH-H and HR2 epitopes.

bioRxiv preprint doi: https://doi.org/10.1101/2021.12.01.470697; this version posted December 3, 2021. The copyright holder for this preprint (which was not certified by peer review) is the author/funder. This article is a US Government work. It is not subject to copyright under 17 USC 105 and is also made available for use under a CC0 license.

S2 Fig: Enrichment of wildtype peptides in baseline macaque samples compared to post-vaccination or post-infection samples. The x axis indicates each peptide's location along SARS-CoV-2 Spike protein, and each entry on the y axis is an individual sample. Sample groups are indicated on the left. The same macaques that contributed baseline samples also contributed post-vaccination or post-infection samples. All enrichment values over 20 are plotted as 20 to better show the lower range of the data. Above the heatmap, domains of Spike are shown with grey boxes, with the S1/S2 and S2' cleavage sites indicated with arrows. The epitope regions defined in the current study are shown as colored boxes (from left to right: NTD in red, CTD-N' in green, CTD-C' in cyan, pre-FP in pink, FP in black, post-FP in orange, SH-H in purple, and HR2 in blue).

S3 Fig: Differences in enrichment of wildtype peptides by macaque subgroups. Wildtype enrichment values were summed for all peptides within each region of Spike that showed enrichment. Each point represents an individual macaque. No significant differences were found by Kruskal-Wallis test at a threshold of p=0.05. LION: Lipid InOrganic Nanoparticle; NLC: Nanostructured Lipid Carrier.

S4 Fig: Use of optimal transport to quantify similarity between amino acid escape profiles. (A) Profile 1 and 2 show example logo plots for two samples across the same region. Negative scaled differential selection values represent mutations that reduce antibody binding. Amino acids of the same color indicate similar chemistry (e.g., green = polar). (B) At each location (in this example, the boxed site in panel A), the profiles are represented as binned distributions where each bin corresponds to the contribution to escape for an amino acid substitution. (C) The optimal transport solution to transform one profile to the other is computed, where the

bioRxiv preprint doi: https://doi.org/10.1101/2021.12.01.470697; this version posted December 3, 2021. The copyright holder for this preprint (which was not certified by peer review) is the author/funder. This article is a US Government work. It is not subject to copyright under 17 USC 105 and is also made available for use under a CC0 license.

cost to "exchange" an amino acid contribution in Profile 1 to an amino acid contribution in Profile 2 is derived from the BLOSUM62 matrix. For the purposes of the schematic, the number of dollar signs associated with each line denotes the relative cost of each move (i.e., more dollar signs = more costly = moving between amino acids that are less similar). (D) To quantify similarity between profiles, an escape similarity score is calculated as the inverse of the total cost to perform the transformation. For more details, see https://matsengrp.github.io/phippery/esc-prof.html. Created with BioRender.com.

S5 Fig. Logo plots for all vaccinated macaques, vaccinated humans, and convalescent macaques in the CTD-N’ epitope region.

S6 Fig. Logo plots for all vaccinated humans, convalescent macaques, and convalescent humans in the FP epitope region.

S7 Fig. Logo plots for all vaccinated macaques, vaccinated humans, convalescent macaques, and convalescent humans in the SH-H epitope region.

S8 Fig. Logo plots for all convalescent macaques in the pre-FP and post-FP epitope regions.

S9 Fig: Differences in enrichment of wildtype peptides in vaccinated humans and convalescent macaques. As in Fig 2, wildtype enrichment values were summed for all peptides within each epitope region of Spike. Multiple Mann-Whitney U tests were performed, with p values corrected for the number of comparisons (8) using the Bonferroni-Dunn method. ****, $p \leq 0.0001$; ***, $p \leq 0.001$; **, $p \leq 0.01$; *, $p \leq 0.05$.
